# MiR-451 is decreased in hypertrophic cardiomyopathy and regulates autophagy by targeting TSC1

**DOI:** 10.1111/jcmm.12380

**Published:** 2014-09-11

**Authors:** Lei Song, Ming Su, Shuiyun Wang, Yubao Zou, Xiaojian Wang, Yilu Wang, Hongli Cui, Peng Zhao, Rutai Hui, Jizheng Wang

**Affiliations:** a State Key Laboratory of Cardiovascular Diseases, Fuwai Hospital, National Center for Cardiovascular Disease, Chinese Academy of Medical Sciences and Peking Union Medical CollegeBeijing, China; b Department of Cardiovascular Surgery, Fuwai Hospital, National Center for Cardiovascular Diseases, Chinese Academy of Medical Sciences and Peking Union Medical CollegeBeijing, China; c China Meitan General HospitalBeijing, China; d Department of Pathology, Affiliated Hospital of Qingdao University Medical CollegeQingdao, China

**Keywords:** hypertrophic cardiomyopathy, microRNA-451, tuberous sclerosis complex 1, autophagy, microRNAs microarray

## Abstract

The molecular mechanisms that drive the development of cardiac hypertrophy in hypertrophic cardiomyopathy (HCM) remain elusive. Accumulated evidence suggests that microRNAs are essential regulators of cardiac remodelling. We have been suggested that microRNAs could play a role in the process of HCM. To uncover which microRNAs were changed in their expression, microRNA microarrays were performed on heart tissue from HCM patients (*n* = 7) and from healthy donors (*n* = 5). Among the 13 microRNAs that were differentially expressed in HCM, miR-451 was the most down-regulated. Ectopic overexpression of miR-451 in neonatal rat cardiomyocytes (NRCM) decreased the cell size, whereas knockdown of endogenous miR-451 increased the cell surface area. Luciferase reporter assay analyses demonstrated that tuberous sclerosis complex 1 (TSC1) was a direct target of miR-451. Overexpression of miR-451 in both HeLa cells and NRCM suppressed the expression of TSC1. Furthermore, TSC1 was significantly up-regulated in HCM myocardia, which correlated with the decreased levels of miR-451. As TSC1 is a known positive regulator of autophagy, we examined the role of miR-451 in the regulation of autophagy. Overexpression of miR-451 *in vitro* inhibited the formation of the autophagosome. Conversely, miR-451 knockdown accelerated autophagosome formation. Consistently, an increased number of autophagosomes was observed in HCM myocardia, accompanied by up-regulated autophagy markers, and the lipidated form of LC3 and Beclin-1. Taken together, our findings indicate that miR-451 regulates cardiac hypertrophy and cardiac autophagy by targeting TSC1. The down-regulation of miR-451 may contribute to the development of HCM and may be a potential therapeutic target for this disease.

## Introduction

Hypertrophic cardiomyopathy (HCM) is a common monogenetic disease of myocardium with an estimated prevalence of 0.2% in the general population 1. HCM is characterized by unexplained cardiac hypertrophy, myocyte disarray and interstitial fibrosis and is an important cause of sudden cardiac death and heart failure 2–4. This disease is mostly caused by mutations in genes encoding sarcomere proteins 5–11. However, the molecular mechanisms that drive the expression of HCM phenotypes are still poorly understood.

MicroRNAs are endogenous non-coding small RNAs that usually act as repressors of target genes by either inhibiting translation and/or by promoting degradation of the target mRNAs. Previous studies have shown that a number of microRNAs play critical roles in controlling cardiac homoeostasis and pathological remodelling, and suggest that microRNAs may serve as potential therapeutic targets for cardiac diseases 12–18. However, the roles of microRNAs in HCM remain unclear. In the present study, we identified the differently expressed microRNAs in HCM by using a microRNAs microarray, and found that miR-451 was one of the most down-regulated microRNAs. The functional roles of miR-451 in cardiac hypertrophy were investigated.

## Materials and methods

### Ethics statements

The human study protocol was approved by the Ethics Committee of Fuwai Hospital (Beijing, China) in accordance with the Helsinki declaration. The written informed consent from all participants was obtained. All animal studies were approved by the Ethics Committee for Animal Study of Fuwai Hospital (Beijing, China).

### Tissues

Left ventricle tissues were obtained from 16 hypertrophic obstructive cardiomyopathy patients who underwent a Morrow septal myectomy from 2011 to 2013 at Fuwai Hospital, Beijing, China. Eight non-hypertrophic cardiomyopathy (NCM) cardiac tissues were obtained from healthy donors died from accident. All of the donors have no history of cardiac disease. The written informed consents were obtained from their relatives. All of the samples for RNA and protein analysis were immediately stored in liquid nitrogen until use. Tissues for transmission electron microscopy analysis were cut and fixed with 2.5% neutral glutaraldehyde at 4°C. The characteristics of healthy myocardium donors and HCM patients are listed in Tables S1 and S2.

### MicroRNAs microarray assay

Total RNA, including the microRNAs, was extracted with the miRNeasy Mini Kit (Qiagen, Hilden, Germany) according to the manufacturer’s instructions. For the microRNAs microarray assay, seven HCM patients and five NCM donors were included. The microRNA expression profiles were determined at the Shanghai Biotechnology Corporation (Shanghai, China) by using the Agilent human miRNA (8*60K) Version16.0 (Agilent Technologies, Santa Clara, CA, USA). Each microarray chip was hybridized with a single sample labelled with cyanine-3 (Cy3). After hybridization, the slides were scanned using an Agilent Microarray Scanner (Agilent Technologies), and the images were converted into intensity values using Feature Extraction software 10.7 (Agilent Technologies). After background subtraction, the data were imported into Gene Spring software 11.0 (Agilent Technologies) for quantile normalization. The mean normalized signal from biological replicates was used for differential expression analysis. MicroRNAs that exhibited a difference in expression levels of at least twofold (*P* < 0.01) between groups were selected for further analysis.

### Quantitative RT-PCR analysis

For the quantitative detection of mature miR-451, total RNA was reverse transcribed using High Capacity cDNA Reverse Transcription Kit (Life Technologies,Grand Island, NY, USA). qRT-PCR was performed with the TaqMan miRNA assay kit (Life Technologies) according to the manufacturer’s protocol. The samples were run on an Applied Biosystems 7500 Fast Real-Time PCR Systems (Applied Biosystems). Thermal reaction cycles of 94°C for 30 sec., and 40 repetitions of 94°C for 5 sec. and 60°C for 30 sec. were used. The levels of U6 snRNA were used to normalize the expression levels.

### Cell culture and transfection

Protocols for primary neonatal rat cardiomyocytes were followed as previously described 18. In brief, newborn Wistar rats were killed and their ventricles were washed and minced in PBS. The tissues were digested in PBS containing 0.06% collagenase (Worthington Biochemical Corporation, Lakewood, NJ, USA) at 37°C. After dissociation, the cells were centrifuged at 800 × g for 5 min. and re-suspended in DMEM with 10% foetal bovine serum (FBS). For differential adhesion, the cells were maintained in an atmosphere of 5% CO_2_ at 37°C for 90 min. The suspension cells were collected, centrifuged and re-suspended in DMEM supplemented with 10% FBS and 0.1 mM bromodeoxyuridine. The cells were then plated and incubated for 24 hrs before treatment. H9c2 and HeLa cells were purchased from the National Platform of Experimental Cell Resources for Sci-Tech (Beijing, China) and cultured in DMEM with 10% FBS in an atmosphere of 5% CO_2_ at 37°C.

For transfection, cells were starved for 24 hrs, and miR-451 mimics or antagomir was transfected into cells with Lipofectamine 2000 (Life Technologies) for overexpression or knockdown of miR-451, according to the manufacturer’s instructions. The final concentration of miRNA mimics for transfection was 100 nM.

### Cell surface area measurement

The neonatal rat cardiomyocytes were plated at a density of 5 × 10^5^ cells/ml and fixed with 4% paraformaldehyde after transfection for 48 hrs. The cells were washed with PBS twice and permeabilized with Triton X-100 in PBS for 5 min. The cells were then washed with PBS and stained with Texas Red-phalloidin (Life Technologies) for 30 min. at room temperature. After washing with PBS, the cells were incubated with 4′,6-diamidino-2-phenylindole (0.1 μg/ml) for 3 min. The cells were washed with PBS and observed using a fluorescence microscope. The cell sizes in each group were assessed using Image Pro-Plus 6.0 software (Media Cybernetics, Bethesda, MD, USA) by analysing 100 cardiomyocytes from at least 10 random fields, as described previously 18.

### Dual luciferase assay

The 3′UTR of human tuberous sclerosis complex 1 (TSC1) containing miR-451 target sites was amplified using the following primers: forward, 5′-AGGACTAGTGGAATGATGGTCAATCAGTG-3′; reverse, 5′-AGGGAGCTCCACAGTGCCAGCTCCAG-3′. The amplified fragment was cloned into the *Spe*I/*Hind*III sites of the pMIR-REPORT vector (Life Technologies) to generate wild-type pMIR-TSC1-3′UTR reporter as described previously 18. The mutant fragment with substitution of miR-451 target site were synthesized (GenScript, Nanjing, China) and cloned into the same vector to generate a mutated pMIR-TSC1-3′UTR plasmid. Both of the wild-type and mutant constructs were verified by sequencing. H9c2 cells were cotransfected with 0.5 μg wild type or mutant constructs, 50 pmol microRNA mimics or scrambled microRNA and 0.01 μg pRL-TK with Lipofectamine 2000 (Life Technologies). The cells were washed and lysed with Passive Lysis buffer (Promega, Madison, WI, USA) at 48 hrs post transfection. The cell lysates were collected for detecting luciferase activity by using the Dual Luciferase Reporter Assay Kit (Promega). The luciferase activity was measured with a luminometer (SIRIUS, Pforzheim, Germany). This experiment was repeated three times.

### Protein extraction and Western blotting

Tissue and cell proteins were lysed, quantified and loaded onto SDS polyacrylamide gels for electrophoretic separation. The proteins were then blotted onto a nitrocellulose membrane. Subsequently, the membrane was blocked with 5% nonfat milk or 2% bovine serum albumin at room temperature before incubation with specific primary antibodies targeted against the following proteins: TSC1, Bcl-2, Beclin-1, GAPDH (Cell Signaling Technology, Beverly, MA, USA) or microtubule-associated protein 1 light chain 3 (LC3) (Sigma-Aldrich, St. Louis, MO, USA). Subsequently, the membrane was incubated with HRP-conjugated secondary antibodies for 1 hr at room temperature, and specific bands were detected with the SuperSignal West Femto Maximum Sensitivity Substrate (Pierce, Rockford, IL, USA). The band intensities were quantified using Quantity One software V4.6.2 (Bio-Rad, Hercules, CA, USA).

### Subcellular location of EGFP-LC3

The plasmid encoding the recombinant EGFP-LC3 protein was kindly provided by Dr. Xuejun Jiang. HeLa cells were transfected with EGFP-LC3, together with miR-451, scrambled microRNA, miR-451 antagomir-451 or control antagomir, for 48 hrs and EGFP-LC3 punctae were observed and analysed with FV1000 confocal microscopy (Olympus, Tokyo, Japan). The number of EGFP-LC3 punctae per cell was quantified. At least 100 cells were included for quantification per each group.

### Transmission electron microscopy analysis

Left ventricle tissues from HCM patients were fixed in 2.5% neutral glutaraldehyde at 4°C and post-fixed in 1% osmium tetroxide in 0.1 M sodium cacodylate buffer with 0.3% potassium ferrocyanide. The samples in each group were stained with 4% uranyl acetate, dehydrated, infiltrated and embedded in epoxy resin. Ultrathin sections were observed with Hitachi H7650 transmission electron microscope (Hitachi, Tokyo, Japan).

### Statistical analysis

All values are presented as means ± SD. The two-tailed Student’s *t*-test was performed with the IBM SPSS statistics 19 software (SPSS Inc., Chicago, IL, USA) to determine the statistical significance of the results between two groups. *P* < 0.05 was considered as statistically significant.

## Results

### MiR-451 is down-regulated in hearts from HCM patients

**Figure 1:**
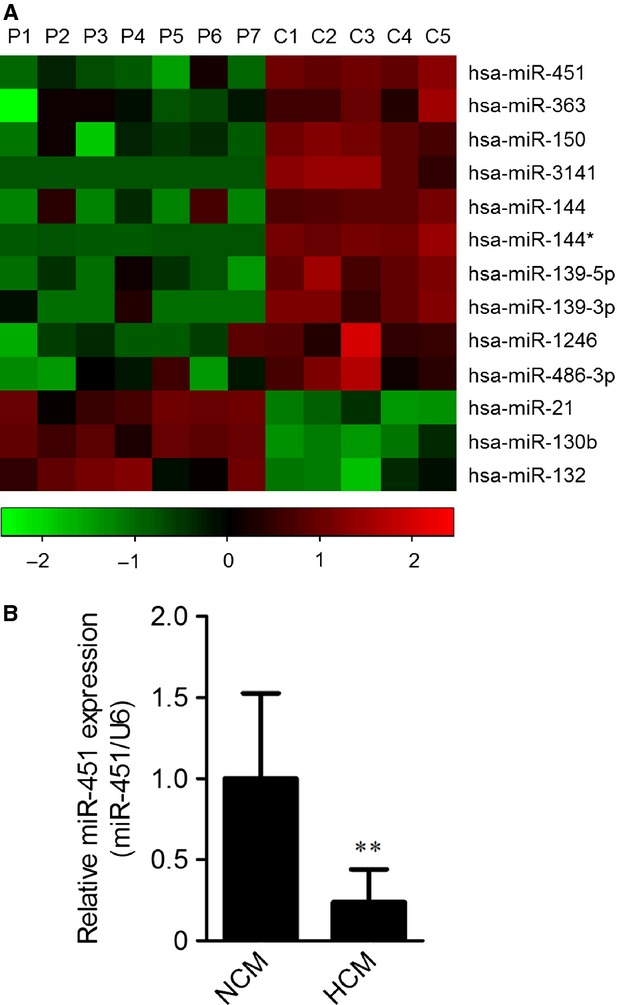
The expression of miR-451 is decreased in hearts from patients with hypertrophic cardiomyopathy. (**A**) Heat map diagram of 13 significantly changed microRNAs in hearts from seven patients (P1–P7) with hypertrophic cardiomyopathy (HCM) and five non-hypertrophic cardiomyopathy (NCM) controls (C1–C5). Red in the colour bar indicates higher expression and green indicates lower expression. (**B**) Relative expressions of miR-451 (normalized to U6) in heart tissues from HCM patients (*n* = 16) and NCM donors (*n* = 8). Data are expressed as means ± SD. **P < 0.01 compared with the NCM group.

To identify differently expressed microRNAs between NCM and HCM hearts, a microarray assay was performed on specimens from five NCM donors and seven HCM patients. In total, three microRNAs (miR-21, miR-130b and miR-132) were significantly up-regulated (≥2-fold, *P* < 0.01), whereas 10 down-regulated (≤0.5-fold, *P* < 0.01) in hearts from HCM patients (Fig. 1A). Among these differently expressed microRNAs, miR-451 ranked as the most down-regulated. To validate the under-expression of miR-451, a qRT-PCR assay was performed with expanded tissue samples (NCM, *n* = 8 and HCM, *n* = 16). As shown in Figure 1B, the expression of miR-451 had decreased more than fourfold.

### MiR-451 regulates cell surface area *in vitro*

**Figure 2:**
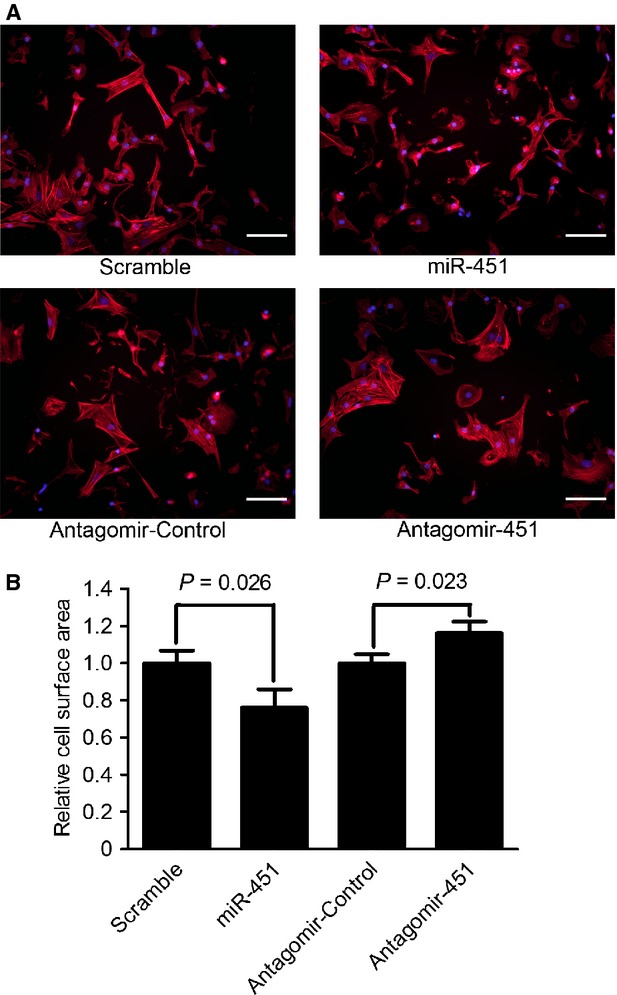
MiR-451 regulates cell size *in vitro*. (**A** and **B**) Primary neonatal cardiomyocytes were transfected as indicated for 48 hrs. Cellular F-actin and the nuclei of the cell in each group were stained with Texas Red-phalloidin and DAPI, respectively; scale bars: 50 μm (**A**). The cell surface area was quantified using Image Pro-Plus 6.0 from at least 100 cells per group (**B**). Data were obtained from three independent experiments, and values are expressed as means ± SD.

MiR-451 was reported to show cardioprotective roles in response to ischaemia/reperfusion stresses 19,20, but its role remains unclear in cardiac hypertrophy. To analyse the effects of miR-451 on cardiac hypertrophy, we transfected primary neonatal rat cardiomyocytes with miR-451 mimics or miR-451 antagomir for 48 hrs after which the cell surface areas were analysed. The surface area of miR-451 overexpressed cardiomyocytes was significantly decreased to 76.0% of the scramble group (*P* < 0.05; Fig. 2). Conversely, the surface area of cardiomyocytes transfected with miR-451 antagomir was increased to 1.17-fold compared with the antagomir control group (*P* < 0.05; Fig. 2).

### TSC1 is a novel target of miR-451

**Figure 3:**
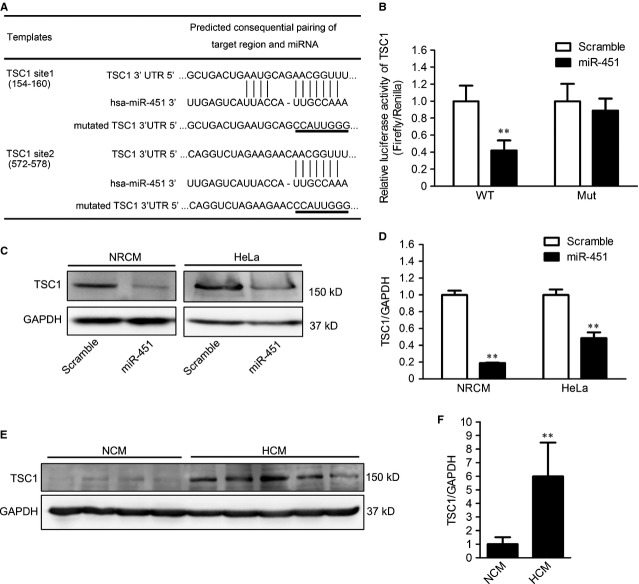
MiR-451 directly targets tuberous sclerosis complex 1 in cardiomyocytes. (**A**) Tuberous sclerosis complex 1 (TSC1) is a potential target gene of miR-451 predicted by targetscan. The binding sites of miR-451 in wild-type (WT) and mutated (Mut) 3′UTR sequences of TSC1 are shown. (**B**) The WT and Mut 3′UTR of TSC1 were cloned into pMIR-REPORT vectors and cotransfected with pRL-TK, miR-451 mimics or scrambled control into H9c2 cells, respectively. The relative luciferase activity was measured and normalized to Renilla. (**C** and **D**) The expression of TSC1 in neonatal rat cardiomyocytes (NRCM) and HeLa cells transfected with miR-451 mimics or scrambled control (**C**). The relative expression of TSC1 was normalized with GAPDH (**D**). (**E** and **F**) The expression of TSC1 from 16 hypertrophic cardiomyopathy (HCM) patients and eight non-hypertrophic cardiomyopathy (NCM) donors, and representative graphs are shown (**E**). The expression of TSC1 was normalized with GAPDH. Values are expressed as means ± SD; *P < 0.05 and **P < 0.01 compared with scrambled control or NCM.

To further investigate the mechanism by which miR-451 regulates cardiac hypertrophy, we searched for the targets of miR-451 by using the TargetScan database. Bioinformatic analysis predicted that TSC1 was a novel target of miR-451. The 3′UTR regions of TSC1 contained two sites complimentary to miR-451 (Fig. 3A). To test whether the predicted TSC1 was a functional target of miR-451, dual luciferase assays were performed. H9c2 cells were transfected with the wild-type pMIR-TSC1-3′UTR or mutated pMIR-TSC1-3′UTR plasmids and cotransfected with either miR-451 mimics or scrambled microRNA. Compared to scrambled microRNA, the miR-451 mimics significantly reduced the luciferase activity of wild-type pMIR-TSC1-3′UTR but did not alter the luciferase activity of mutated pMIR-TSC1-3′UTR (Fig. 3B).

We next assessed whether miR-451 suppressed the expression of endogenous TSC1. Primary neonatal rat cardiomyocytes and HeLa cells were transfected with miR-451 mimics or scrambled microRNA. After 48 hrs, the expression of TCS1 was measured using Western blotting. As expected, the expression of TSC1 was markedly down-regulated by overexpression of miR-451 in both neonatal cardiomyocytes and HeLa cells (Fig. 3C and D).

Considering the expression of miR-451 was down-regulated in the hearts from HCM patients, we have been suggested that the expression of TSC1 could be up-regulated in HCM hearts. To test this, we conducted Western blotting to detect the expression of TSC1 in hearts from 16 HCM patients and eight NCM donors. Consistent with the *in vitro* results, increased expression of TSC1 was observed in HCM hearts (Fig. 3E and F).

### Autophagy is inhibited by miR-451

**Figure 4:**
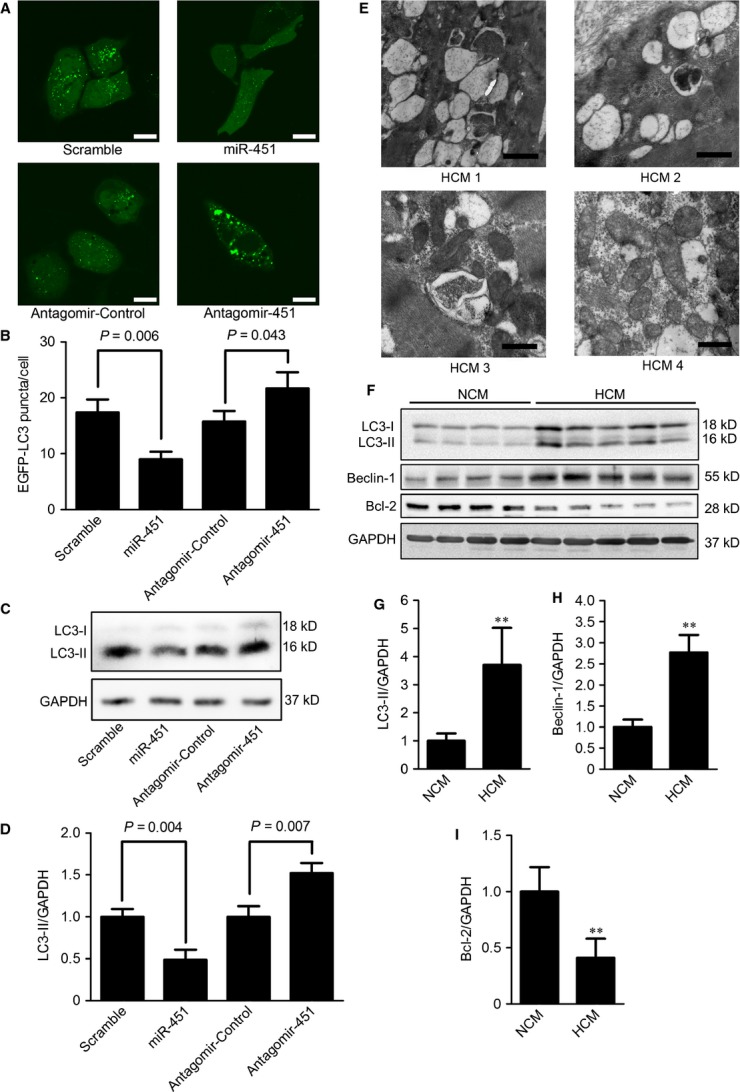
MiR-451 activates autophagy in cardiomyocytes. (**A** and **B**) HeLa cells were transfected with plasmid encoding EGFP-LC3, together with miR-451 mimics, scrambled microRNA, antagomir-451 or antagomir-control. Representative images (**A**) and quantification (**B**) of EGFP-LC3 punctae (green) are shown; scale bars: 20 μm. (**C** and **D**) Lipidation levels of LC3 in neonatal rat cardiomyocytes transfected with miR-451 mimics, scrambled control, antagomir-451 or antagomir control. (**E**) The ultrastructure of cardiomyocytes from four patients with hypertrophic cardiomyopathy (HCM); scale bars: 0.5 μm. (**F**–**I**) Western blot analysis for the expression of LC3, Beclin-1 and Bcl-2 in hearts from 12 HCM patients and eight non-hypertrophic cardiomyopathy (NCM) donors. Representative graphs are shown (**F**). Densitometry analysis of LC3-II (**G**), Beclin-1 (**H**) and Bcl-2 (**I**) were normalized with GAPDH. Values are expressed as means ± SD; **P < 0.01 compared with NCM.

As TSC1 is expressed in various cardiovascular tissues and activates autophagy 4,21,22, we assessed whether autophagy was regulated by miR-451. An autophagy-reporter plasmid encoding EGFP-LC3 recombinant protein was cotransfected with miR-451 mimics or antagomir into HeLa cells. The formation of autophagosomes was observed based on the appearance of EGFP-LC3 punctae. We found that HeLa cells transfected with miR-451 mimics had a significantly lower number of EGFP-LC3 punctae compared with those transfected with scrambled microRNAs. Conversely, the suppression of miR-451 in HeLa cells resulted in larger amounts of EGFP-LC3 punctae (Fig. 4A and B).

We next evaluated the effect of miR-451 on autophagy in neonatal rat cardiomyocytes. After transfection with miR-451 mimics or miR-451 antagomir, the autophagy level was determined by measuring the autophagy marker, the lipidated form of LC3 (LC3-II), which is closely related with the formation of autophagosomes 23. We observed that the LC3-II in cells with miR-451 overexpression was significantly decreased, whereas cells transfected with miR-451 antagomir showed increased LC3-II (Fig. 4C and D).

### Increased autophagy in the hearts from HCM patients

The status of autophagy in cardiac tissues of HCM remains unclear. Transmission electron microscopy showed that the sarcomeric filaments in HCM cardiomyocytes were disrupted and sparse (Fig. 4E). Furthermore, we observed that large quantities of early and late autophagic vacuoles with cytoplasmic remnants and mitochondria had accumulated in the cytosol of cardiomyocytes from HCM hearts (Fig. 4E).

We further determined the expression levels of autophagic markers in heart tissues from HCM patients. The levels of LC3-II were dramatically increased in hearts from HCM patients (Fig. 4F and G). The expression of Beclin-1, a required protein in membrane nucleation during the early stage of autophagy, was also significantly up-regulated in HCM hearts, whereas Bcl-2 protein, an inhibitor of autophagy, is down-regulated (Fig. 4F, H and I).

## Discussion

MicroRNAs play crucial roles in regulating cardiac remodelling; however, many of them have not been well characterized in HCM. In this study, we found that miR-451 was markedly down-regulated in heart tissues from HCM patients. Secondly, we elucidated that TSC-1 was a novel functional target responsible for miR-451 in cardiomyocytes. Finally, our study unveiled that miR-451 negatively regulated cardiac hypertrophy and cardiac autophagy *via* targeting TSC1.

Previous studies suggest that the role of miR-451 in the heart may be protective in response to stresses. Ectopic expression of miR-451 protects cardiomyocytes from ischaemia/reperfusion injury, whereas knockdown of miR-451 exacerbates any simulated ischaemia/reperfusion-induced cell death 20. Wang *et al*. reported that genetic knockout of miR-451 impairs ischaemic preconditioning-mediated cardioprotection exposed to ischaemic/reperfusion injury 19. In the present study, we scanned the expression of microRNAs using a microRNAs microarray. We found 13 significantly differentially expressed microRNAs that may associate with HCM. Our microRNA profiling analysis indicated that miR-451 was one of the most down-regulated microRNAs in HCM patients, which was confirmed by qRT-PCR. Overexpression of miR-451 in neonatal rat cardiomyocytes decreased cell size, whereas knockdown of endogenous miR-451 induced cardiac hypertrophy. These results indicated that the down-regulation of miR-451 might be involved in the development of HCM.

Previous studies reported that dysregulated autophagy contributes to the development of cardiac remodelling 24–26, but little is known about the alteration of autophagy in HCM. Autophagy is a bulk protein degradation process that is responsible for the degradation of long-lived proteins and cytoplasmic organelles. Cardiac autophagy is responsible for the turnover of organelles at basal levels under physical conditions, however, it can be activated in response to stresses 27. The balance of normal autophagy levels is required to maintain cardiac homoeostasis 28. In contrast, excessive autophagy activation accentuates pathological remodelling 29,30. In an endomyocardial biopsy of a patient in transition from HCM to heart failure, large numbers of autophagic vacuoles were observed 31, suggesting that autophagy is activated in the late stage of HCM hearts. Consistently, we observed that autophagosomes had accumulated in cardiomyocytes from HCM patients without heart failure. Similarly, the levels of LC3-II and Beclin-1 were both increased in HCM tissues from a myocardiectomy. Thus, our results indicate that autophagy is activated in stable hypertrophic tissues from HCM patients.

Previous studies reported that miR-451 inhibited the activity of AMPK by down-regulating LKB1 32,33. AMPK is a known autophagy-promoting factor by activating the TSC1-TSC2 complex 34–37. However, we found that the activity of AMPK was not altered by either overexpression or knockdown of miR-451 in cultured neonatal rat cardiomyocytes (data not shown). In most of the myocardia from HCM patients where miR-451 was significantly down-regulated, the activity of AMPK tended to be decreased (data not shown). Thus, AMPK may not play a crucial role among the regulation of autophagy by miR-451 in cardiomyocytes.

Tuberous sclerosis complex 1 is a crucial regulator of autophagy by forming a heterodimer with TSC2. Overexpression of TSC1 activates autophagy, whereas depletion of TSC1 suppresses autophagy 38,39. In the present study, we demonstrated that TSC1 was a direct target of miR-451. MiR-451 negatively regulated the expression of endogenous TSC1 and autophagy. Consistently, in HCM myocardia, where the expression of miR-451 was significantly decreased, up-regulated TSC1 and activated autophagy were observed. These data demonstrate that the miR-451 plays an important role in regulating cardiac autophagy potentially by the targeted suppression of the expression of TSC1.

In summary, our study reveals that miR-451 is one of the most down-regulated microRNAs in HCM and regulates cardiac hypertrophy and cardiac autophagy by targeting TSC1. The down-regulation of miR-451 may contribute to the development of HCM and may be a potential therapeutic target for the disease.

## Supporting Information

**Table S1.** Characteristics of healthy myocardium donors. **Table S2.** Characteristics of patients with hypertrophic cardiomyopathy.
