# Supplementary material for: MiR-451 is decreased in hypertrophic cardiomyopathy and regulates autophagy by targeting TSC1
Source: J Cell Mol Med. 2014 Sep 11;18(11):2266–74. doi: 10.1111/jcmm.12380 (PMC4224559; doi:10.1111/jcmm.12380)
Supplement: Supplementary file 1 — Table S1. Characteristics of healthy myocardium donors. Table S2. Characteristics of patients with hypertrophic cardiomyopathy. [file jcmm0018-2266-sd1.docx]

Supplementary Table 1 Characteristics of healthy myocardium donors

| ID | Age (year) | Gender | MicroRNAs microarray | qRT-PCR | Western blot |
| --- | --- | --- | --- | --- | --- |
| C1 | 21 | male | √ | √ | √ |
| C2 | 27 | male | √ | √ | √ |
| C3 | 24 | male | √ | √ | √ |
| C4 | 29 | male | √ | √ | √ |
| C5 | 27 | male | √ | √ | √ |
| C6 | 23 | male | ND | √ | √ |
| C7 | 26 | female | ND | √ | √ |
| C8 | 30 | male | ND | √ | √ |

√: detected; ND: not detected

Supplementary Table 2 Characteristics of patients with hypertrophic cardiomyopathy

| ID | Age (year) | Gender | IVST (mm) | LVOTPG (mmHg) | Mutation | MicroRNAs microarray | TEM | qRT-PCR | Western blot |
| --- | --- | --- | --- | --- | --- | --- | --- | --- | --- |
| HCM1038 | 38 | male | 18 | 145 | MYH7: 1477I and R1606C | √ | ND | √ | √ |
| HCM1046 | 42 | male | 29 | 112 | MYH7: F489C | √ | ND | √ | √ |
| HCM1056 | 34 | male | 26 | 34 | MYH7: A355T | √ | ND | √ | √ |
| HCM1059 | 37 | male | 30 | 61 | UD | √ | ND | √ | √ |
| HCM1060 | 15 | male | 34 | 64 | MYH7: R403L | √ | ND | √ | √ |
| HCM1062 | 60 | male | 26 | 67 | MYH7: R663C | √ | ND | √ | √ |
| HCM1066 | 49 | female | 22 | 100 | UD | √ | ND | √ | √ |
| HCM0787 | 47 | male | 24 | 77 | MYH7: E1356K and MYBPC3: R160W | ND | ND | √ | √ |
| HCM0729 | 60 | female | 19 | 88 | UD | ND | ND | √ | √ |
| HCM1076 | 31 | female | 26 | 90 | MYH7: R663C | ND | ND | √ | √ |
| HCM1078 | 31 | male | 23 | 112 | MYH7: R663H | ND | ND | √ | √ |
| HCM1079 | 48 | female | 20 | 130 | MYBPC3: R773C | ND | ND | √ | √ |
| HCM1080 | 31 | male | 18 | 59 | UD | ND | √ | √ | √ |
| HCM1083 | 26 | male | 35 | 96 | ND | ND | √ | √ | √ |
| HCM1092 | 38 | male | 19 | 71 | MYBPC3: D770N | ND | √ | √ | √ |
| HCM1093 | 45 | male | 38 | 50 | UD | ND | √ | √ | √ |

IVST: interventricular septal thickness; LVOTPG: left ventricular outflow tract pressure gradient; TEM: Transmission electron microscopy analysis; √: detected; UD, disease mutation was undetected in eight sarcomere genes; ND: not detected
